# *C. elegans* as an Animal Model to Study the Intersection of DNA Repair, Aging and Neurodegeneration

**DOI:** 10.3389/fragi.2022.916118

**Published:** 2022-06-22

**Authors:** Francisco José Naranjo-Galindo, Ruixue Ai, Evandro Fei Fang, Hilde Loge Nilsen, Tanima SenGupta

**Affiliations:** ^1^ Department of Clinical Molecular Biology, University of Oslo, Oslo, Norway; ^2^ Section of Clinical Molecular Biology (EpiGen), Akershus University Hospital, Lørenskog, Norway; ^3^ Department of Microbiology, Oslo University Hospital, Oslo, Norway

**Keywords:** aging, neurodegenerative diseases, DNA repair, Parkinson’s disease, *Caenorhabditis elegans*, Alzheimer’s disease

## Abstract

Since its introduction as a genetic model organism, *Caenorhabditis elegans* has yielded insights into the causes of aging. In addition, it has provided a molecular understanding of mechanisms of neurodegeneration, one of the devastating effects of aging. However, *C. elegans* has been less popular as an animal model to investigate DNA repair and genomic instability, which is a major hallmark of aging and also a cause of many rare neurological disorders. This article provides an overview of DNA repair pathways in *C. elegans* and the impact of DNA repair on aging hallmarks, such as mitochondrial dysfunction, telomere maintenance, and autophagy. In addition, we discuss how the combination of biological characteristics, new technical tools, and the potential of following precise phenotypic assays through a natural life-course make *C. elegans* an ideal model organism to study how DNA repair impact neurodegeneration in models of common age-related neurodegenerative diseases.

## Introduction

**FIGURE 1:**
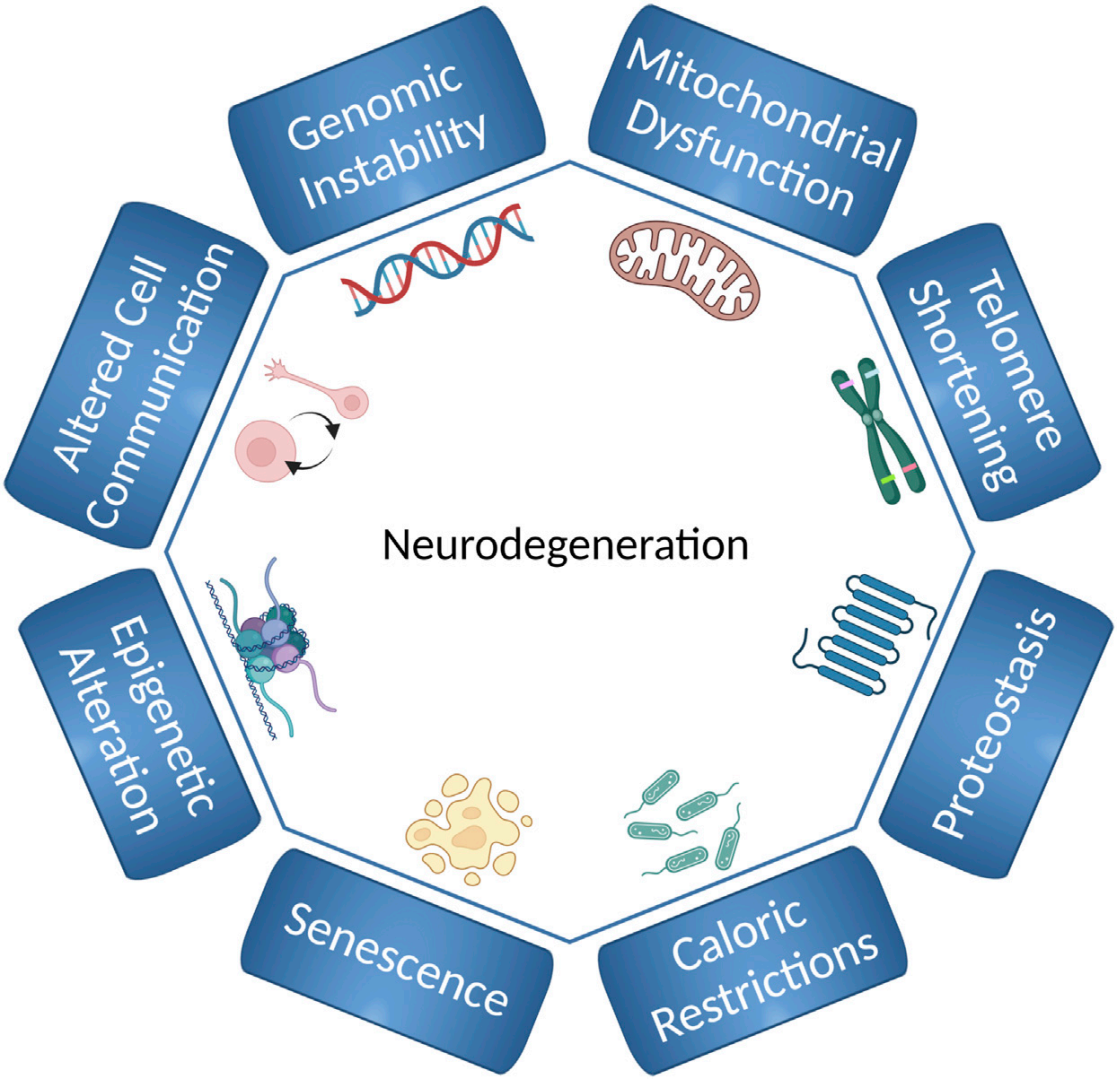
Hallmarks of aging and neurodegeneration in *C. elegans*. The hallmarks of aging and neurodegeneration in *C. elegans* include genomic instability, telomere shortening, epigenetic alterations, loss of proteostasis, deregulated nutrient-sensing, mitochondrial dysfunction, cellular senescence, and altered cell communication. The figure has been generated using Biorender.com by the author.

Neurodegenerative diseases like Parkinson’s disease (PD) and Alzheimer’s disease (AD) present a significant healthcare challenge. Aging is the major risk factor for these neurodegenerative diseases (Fang et al., 2017; Hou et al., 2019). Many cellular processes contribute to aging (Figure 1). These processes are often referred to as the “hallmarks of aging” and include genomic instability, epigenetic alteration, mitochondrial dysfunction, loss of proteostasis, senescence, telomere shortening, altered metabolism and cell-cell communication, stem cell exhaustion and, as recently proposed, compromised autophagy (López-Otín et al., 2013; Hansen et al., 2018; Aman et al., 2021). A major challenge for future research is to understand how these complex processes interact to influence aging. Simple model systems, such as *C. elegans*, remain important tools because they allow us to study the interaction between different mechanisms of aging, and how the aging process leads to development of age-related diseases.

The Nobel Laureate Sydney Brenner established the nematode *C. elegans* as a model system (Brenner, 1974). *C. elegans* is sexually dimorphic, with the majority of the population consisting of self-fertilizing hermaphrodites. Males constitute a small portion of the population (0.1%) (Brenner, 1974). The full genome sequence of this small nematode was completed in 1998 (Consortium*, 1998) and almost 70% of the 19,000 genes are conserved between *C. elegans* and humans (Consortium*, 1998). *C. elegans* develops through a program that takes it through the embryonic stage, four larval stages (L1 through L4) into a reproductive adult in 3 days (at 20°C). In case the environment is not favorable, e.g., due to overpopulation or lack of food, larvae may go into an alternative developmental stage, referred to as the dauer stage, where they become stress-resistant and may survive for several months until they, upon encountering food, go back to the developmental cycle (Riddle et al., 1981).

A large proportion of the 959 post-mitotic somatic cells in *C. elegans* (Sulston and Horvitz, 1977) belong to the nervous system. This includes 302 neurons and 56 glia-like cells, and 7600 synapses (White et al., 1986). Most classical neurotransmitters such as glutamate (Glu), gamma-Aminobutyric acid (GABA), dopamine (DA), serotonin (5-hydroxytryptamine; 5-HT), and acetylcholine (ACh) are present in the worms (Brownlee and Fairweather, 1999). The interactions of *C. elegans* neurons, synapses, and neurotransmitters are similar to those of mammals (Bargmann, 1998). Here, we will discuss how *C. elegans* is used as model system to study various hallmarks of aging and neurodegeneration with a special focus on genomic instability and mitochondrial dysfunction. Additionally, we also discuss limits, recent advancements, and new techniques that can be implemented to study the role of DNA damage as a driver of aging and neurodegeneration.

### *C. elegans* in Aging Research

Aging is the process of gradual functional decline that an organism experiences over time. Because aging is the main risk factor for neurodegenerative diseases (NDs) (Hou et al., 2019), an understanding of the aging processes is highly relevant in a perspective of translational research.

Nematodes were regarded as a preferred model for aging research (Gershon, 1970) due to characteristics like their morphological simplicity and the possibility to follow large populations through a natural short life course. This, combined with Sydney Brenner’s influential article in 1974 highlighting the possibilities of *C. elegans* linking mutations and their phenotypic effects (Brenner, 1974), established the possibility to use *C. elegans* to find modifiers of aging and lifespan (Gershon, 1970). Researchers started describing the aging process in *C. elegans* (Croll et al., 1977; Klass, 1977) and the impact of factors like temperature and food (Klass, 1977) while developing methods for the isolation of mutants with altered longevity (Klass, 1983). Progressively, it was established that aging modified measurable parameters like behavior, chemotaxis or locomotion (Hosono, 1978; Hosono et al., 1980; Johnson, 1987), highlighting how the study of aging implicates more variables than the lifespan itself. The first mutants associated with lifespan extension were subsequently characterized and linked to variants in the *age-1* gene (Friedman and Johnson, 1988). As the field progressed, a new gene was identified as a modifier of lifespan, *daf-2* (Kenyon et al., 1993). Mutants in *daf-2* had lifespan double that of the wild type, a phenotype suppressed by mutations in *daf-16*, that would later be shown to suppress also the long-lived phenotype of *age-1* (Murakami and Johnson, 1996).

Interestingly, these genes had been linked previously with another process that modified lifespan, lifecycle and aging: dauer formation. The screens for genes that modified dauer formation had been done previously (Albert et al., 1981; Riddle et al., 1981), identifying *daf-2* and *age-1* mutants (in previous publications denominated *daf-23*) as dauer constitutive which means that even in favorable growth conditions, a percentage of those populations would still go into the dauer stage; with mutations in *daf-16* suppressing this dauer constitutive phenotype (Vowels and Thomas, 1992). This interplay between AGE-1 and DAF-2*,* with DAF-16 opposing their functions in lifespan and dauer formation, would culminate in a series of publications identifying these genes as the components of the insulin/IGF-1 signaling (IIS) pathway in the nematode (Morris et al., 1996; Morris et al., 1996; Kimura et al., 1997; Lin et al., 1997; Ogg et al., 1997; Tissenbaum and Ruvkun, 1998), a pathway conserved throughout evolution and that regulates lifespan across different organisms (Tissenbaum and Ruvkun, 1998; Tatar et al., 2001; Barbieri et al., 2003; Blüher et al., 2003; Holzenberger et al., 2003; Hwangbo et al., 2004), including humans (Hwangbo et al., 2004; Suh et al., 2008; Willcox et al., 2008).

Later, many different factors have been identified to impact aging using *C. elegans* as a model, including oxidative stress (Larsen, 1993; Vanfleteren, 1993; Park et al., 2009), DNA repair (Hyun et al., 2008; Arczewska et al., 2013; Lans et al., 2013; Fang et al., 2016; SenGupta et al., 2021) and epigenetics (Maures et al., 2011; Li and Casanueva, 2016; Martin-Herranz et al., 2019). Thus, *C. elegans* is established as a model organism for aging. In the following chapters, we highlight some of these processes and how they affect the development of NDs.

### Hallmarks of Aging

Among the known hallmarks of aging (Figure 1), we briefly address some hallmarks that are affected by genomic instability and discuss how in *C. elegans* can be used to study the contribution of these processes to neurodegeneration and aging.

### Mitochondrial Dysfunction

**FIGURE 2:**
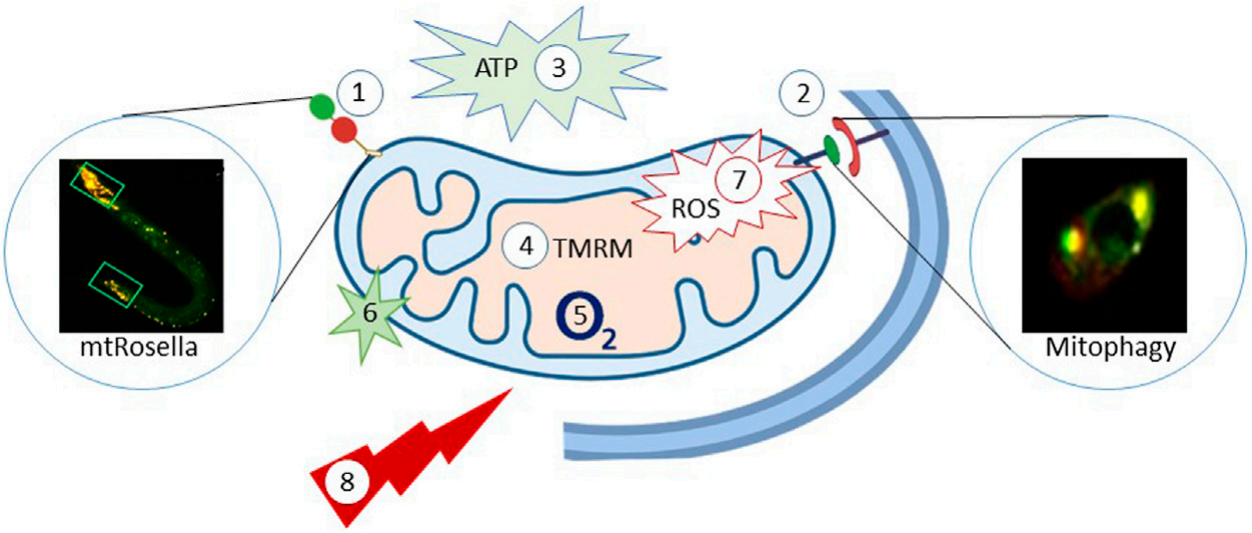
*C. elegans* as model organism to study mitochondrial homeostasis and function. The *C. elegans* provide a way to monitor mitophagy; 1: Mitochondria-target Rosella (mtRosella), using the Rosella biosensor combining a pH-insensitive DsRed and pH-insensitive GFP. 2: Co-localization between DCT-1 (outer mitochondrial membrane protein) and LGG-1 (autophagosomal membrane protein, homolog of the mammalian LC3). 3: Luciferase-based methods to evaluate how different conditions affects the production of ATP in C. elegans; The Roche ATP bioluminescent assay kit HSII is another way to determine ATP content. 4: TMRM: the TMRM staining-tetramethylrhodamine, ethyl ester, perchlorate, a dye that accumulates in intact, respiring mitochondria. 5: Oxygen consumption rate (OCR) is another parameter to reflect mitochondria function. 6: Mito Tracker Green FM is a green-fluorescent dye that stains mitochondria in live cells and its accumulation is dependent on membrane potential. 7: mitochondrial ROS and cellular ROS can be quantification via mtROS (MitoTracker Red CM-H2X ROS); DHE (dihydroethidium), respectively. 8: Stress resistance-survival can also be detected via a heat stress assay, CCCP stress assay, ultraviolet light stress assay, paraquat-induced oxidative stress assay and starvation assays. The figure has been generated using power point image building tool by the author.

Mitochondria support neurons by generating ATP (Mattson et al., 2008) that provides the energy for cellular activities that maintain neuronal function and structure. Mitochondria regulate Ca^2+^ -and redox signaling which impact on synaptic plasticity (Jung et al., 2020). Mitochondrial dysfunction, caused by different reasons, such as mutations in mitochondrial genes and as well as intracellular and extracellular stresses to mitochondria, contributes to aging (Sun et al., 2016) and neurological disorders (Mattson et al., 2008) (Figure 1 and Figure 2). For instance, in animal/cellular models of AD bearing Aβ pathology, higher mitochondria-mediated oxidative stress, impaired Ca^2+^ homeostasis, derailed energy metabolism, and apoptosis were evident (Mattson et al., 2008). Indeed, Aβ directly cause mitochondrial damage, including increased mitochondrial ROS, higher mitochondrial Ca^2+^ uptake, and decreased ATP production (Hashimoto et al., 2003). In PD, mitochondrial complex I activity is decreased resulting in ATP depletion, ROS production, and excitotoxic Ca^2+^ overload. In AD, dysfunction of proteins like α-synuclein, Parkin, DJ-1, PINK1, UCHL1, and LRRK2 result in mitochondrial dysfunction (Lautrup et al., 2019). In a *C. elegans* PD model, mitochondrial fusion and fission defect increases sensitivity of dopaminergic neurons to UVC but makes them resistant to the neurotoxin 6-OHDA (Hartman et al., 2019). In Huntington’s disease (HD), adverse effects on mitochondria have been reported including mitochondrial electron transport impairment (Brouillet et al., 2005), mitochondrial trafficking impairment (Chang et al., 2006), ATP reduction in synaptic terminals (Orr et al., 2008), and mitochondrial depolarization (Panov et al., 2002). In a *C. elegans* model of HD, disruption of the mitochondrial fission gene *drp-1* exacerbates the phenotype, whereas decreasing mitochondrial fragmentation improved protection (Machiela et al., 2021).

Mitochondrial function in *C. elegans* can be measured using several strategies: for example, luciferase-based reporters are available to evaluate oxidative phosphorylation (OXPHOS), glycolysis, and fatty acid oxidation (Luz et al., 2016). The oxygen consumption rate is widely used to study mitochondrial function. ATP levels can also be measured in *C. elegans* (Palikaras and Tavernarakis, 2016). ROS sensitive dyes, like DHE (Dihydroethidium) or Mitotracker Red can be used to detect cellular ROS. Some stress resistance/survival parameters can also reflect mitochondrial function, including assays to stress induced by heat (Zevian and Yanowitz, 2014), mitochondrial uncoupling (Palikaras et al., 2015), ultraviolet light (Park et al., 2017), paraquat and juglone (Senchuk et al., 2017), and starvation (Palikaras et al., 2015).

### Autophagy

Autophagy is a dynamic process dedicated to maintain the cellular homeostasis, normal growth and development (Meléndez et al., 2003). Ample studies have shown that loss of autophagy genes either extend or shorten lifespan in *C. elegans* (Hars et al., 2007; Hashimoto et al., 2009). However, neuronal selective autophagy may have a particular role in the development of neurodegenerative disorders (Conway et al., 2020; Konstantinidis and Tavernarakis, 2021). Selective autophagy of mitochondria, mitophagy, seems to be critical for neuronal health (Aman et al., 2021). Mitophagy functions as a machinery that selectively degrades damaged mitochondria in response to numerous stresses such as starvation and oxidative stress (Palikaras et al., 2018; Pickles et al., 2018). Mitophagy deficiency reduces lifespan and healthspan. In humans and mice, mitophagy levels were shown to be lower in the hippocampus dentate gyrus, hearts, and skeletal muscle satellite cells (Hoshino et al., 2013; Sun et al., 2015; Bravo-San Pedro et al., 2017). Transgenic reporter strains (Figure 2) of mitophagy (Palikaras et al., 2015), has been used to reveal improvements in healthspan and lifespan when mitophagy-related genes, such as mitochondrial fission protein dynamin-related protein 1 (DRP1), Parkin, and PTEN-induced kinase-1 (PINK-1) are up-regulated (Todd and Staveley, 2012; Palikaras et al., 2015; Schiavi et al., 2015).

Deregulation of mitophagy has been linked to several age-related neurodegenerative diseases, including AD (Batlevi and La Spada, 2011), PD (Schapira, 2011), and HD (Batlevi and La Spada, 2011). In AD, for example, mitophagy stimulation restores memory loss via PINK-1, PDR-1 (Parkinson’s disease-related-1; parkin), or DCT-1 (DAF-16/FOXO-controlled germline-tumor-tumor-affecting-1)-dependent pathways (Fang et al., 2019a). Loss of function mutations in mitophagy genes (PINK-1 and PARK2) has been linked to PD (Narendra et al., 2008; Narendra et al., 2010; Hartman et al., 2019).

### Loss of Proteostasis

Protein homeostasis (proteostasis) is well conserved in eukaryotes and functions to maintain the proteome and prevent misfolding and protein aggregation (Santra et al., 2019). Chaperones, the organelle-specific UPR pathway, the ubiquitin-proteasome system (UPS), ERAD and autophagy machinery all contribute to proteostasis (Labbadia and Morimoto, 2015) (Figure 1). In *C. elegans* loss of proteostasis has been linked to aging and cell death as a result of exhaustion, or failure, in chaperone activity (Li and Casanueva, 2016). The mitochondrial UPR (UPRmt) has been shown to be activated when mitochondrial protein import is inhibited, reducing mitochondrial load and subsequently improving longevity in *C. elegans* (Lionaki et al., 2022). The endoplasmic reticulum UPR (UPR^ER^) transcription factor XBP-1 in the neurons may activate intestinal UPR^ER^in *C. elegans* via neuronal signal, leading to improved proteostasis and subsequently improving the lifespan (Imanikia et al., 2019). Nematodes has been widely used to investigate age-related organelle-specific proteostasis failure and its implications for lifespan regulation (Hsu et al., 2003; Meléndez et al., 2003; Morley and Morimoto, 2004; Palikaras et al., 2015). *C. elegans* AD, PD, HD and ALS models have been used to show that proteostasis collapse are associated to a failure in amyloid, tau, α-synuclein, and extended polyQ clearance (Voisine et al., 2010; Lehrbach and Ruvkun, 2019; Ruz et al., 2020) (see below).

### Telomere Shortening and Stem Cell Exhaustion

Telomere length and senescence impact longevity in humans and higher species. Likewise, in *C. elegans*, telomere length is a determinant of longevity and lifespan (Lim et al., 2001; Joeng et al., 2004) (Figure 1). Although the senescence-related secretory phenotype in *C. elegans* is still poorly understood, senescence-like atrophy is responsible for the age-dependent loss of gonad cells in the distal tip of the germline (de la Guardia et al., 2016), as well as insulin/IGF-1 signaling-dependent self-destruction of intestinal biomass (Ezcurra et al., 2018; Kern et al., 2021). This indicates that this model organism might experience age-related senescence, but stem cell exhaustion has not been directly linked to aging in *C. elegans.* However, a growing body of evidence suggests that germline stem cells may have a role in neuronal fate maintenance (Kimble and Seidel, 2008; Tursun et al., 2011; Devanapally et al., 2015; Wu et al., 2015; Marchal and Tursun, 2021), and a new study further substantiates that thermosensory neurons regulate longevity and germline stem cell exhaustion (Lee et al., 2019).

### Genomic Instability

Genomic instability is a key hallmark of aging (Figure 1) (López-Otín et al., 2013). Failure to maintain genome stability, e.g. by defects in DNA repair, is associated with a range of phenotypes, from severe developmental defects to very modest disorders. Some DNA repair diseases display some, but not all, signs of aging—a characteristic referred to as 'segmental progeria' (Rieckher et al., 2021). These syndromes highlight that inefficient DNA repair contributes to aging and neurodegeneration (Keijzers et al., 2017). Importantly, genomic instability may also exacerbate, or even drive, other hallmarks, such as telomere erosion and mitochondrial dysfunction (van der Rijt et al., 2020).

**FIGURE 3:**
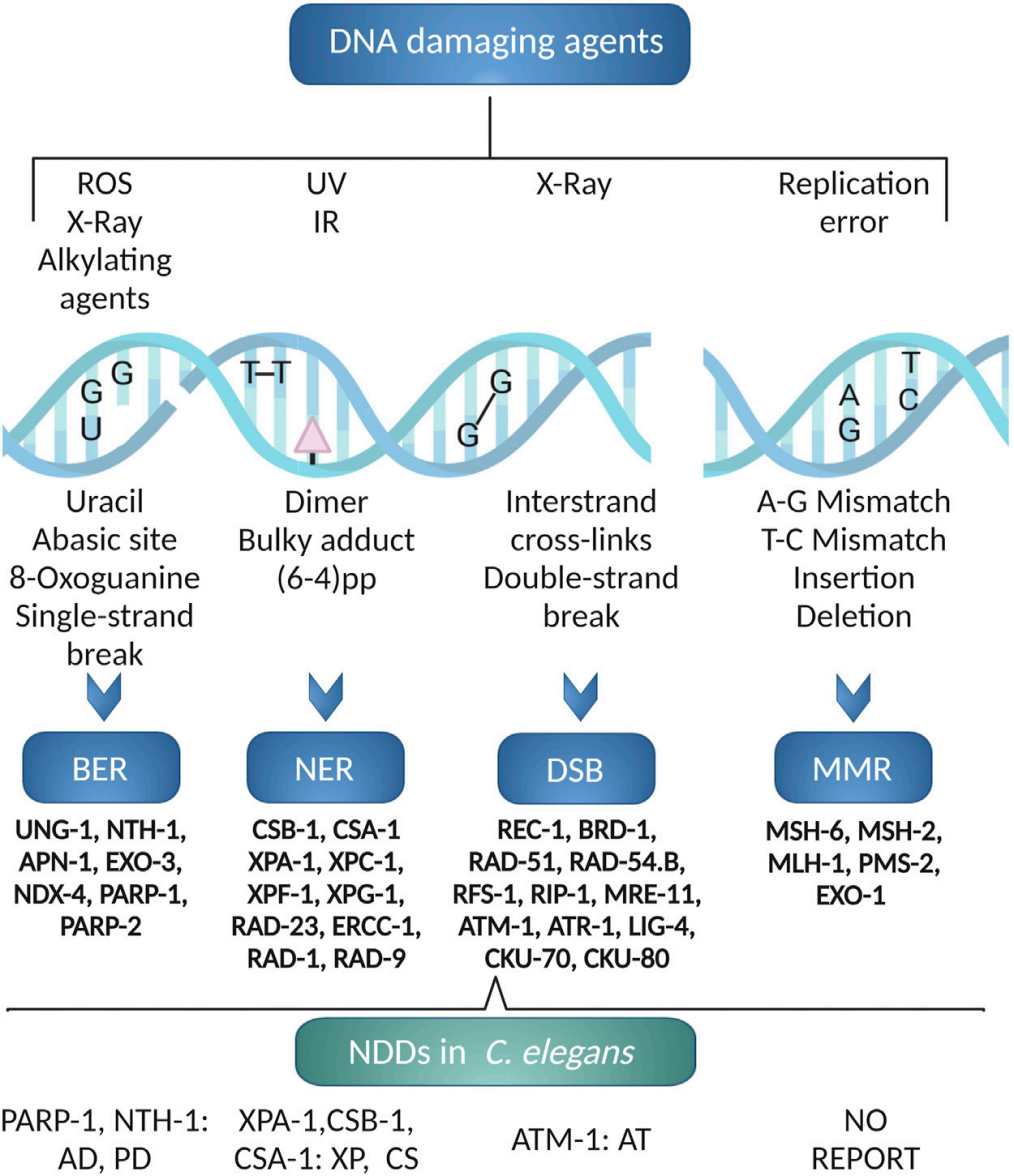
*C. elegans* DNA repair pathways. The excision repair pathways BER, NER, DSB and MMR are operative in *C. elegans*. Using *C. elegans* various neurodegenerative diseases like AD, PD, XP, CS and AT associated to DNA repair defects has been modeled. The figure has been generated using Biorender.com by the author.

The major DNA repair pathways are represented in *C. elegans* and most are well conserved (Rieckher et al., 2018) (Figure 3). Most of our knowledge on DNA repair in *C. elegans* comes from studies of germline genome stability which is the only organ containing proliferating cells in the adult worm (Vermezovic et al., 2012). Germline DNA repair can be followed through phenotypic end-points such as survival assays, brood size, and male frequency as a marker of germline meiotic crossing-over defects (initiated by programmed introduction of double-stranded breaks by SPO-11) (Craig et al., 2012). Immunohistochemistry and fluorescence-based reporters are standardized tools to monitor apoptosis and activation of classical DNA damage response signaling of germline cells (and embryos) (Vermezovic et al., 2012). Direct measurements of nuclear and mitochondrial DNA damage are also possible from whole worm extracts (Arczewska et al., 2013; SenGupta et al., 2021), but as the number of germline nuclei exceeds the somatic cells, such assays will be dominated by germline effects. Similarly, the comet assay can be used to measure DNA single-or double strand breaks in germline nuclei or embryos (Park et al., 2016). Methods to visualize DNA damage response markers in somatic cells are still lacking although, immunohistochemical analyses has been used to detect single-stranded breaks (SenGupta et al., 2021).

Mature neurons are post mitotic. The DNA repair pathways that are not primarily coupled to DNA replication, that is base excision repair (BER), nucleotide excision repair (NER) and non-homologous end joining (NHEJ) (McKinnon, 2009), are therefore likely to be more important for their maintenance. NER is a versatile pathway needed for repair of covalent helix-distorting adducts that has been extensively studied in *C. elegans.* Both transcription-coupled (TC-NER) and global genome (GG-NER) NER pathways are active (Figure 3). In response to UV-induced DNA damage, germline cells rely primarily on GG-NER; TC-NER serves as a backup pathway when GG-NER is not present (Lans and Vermeulen, 2011). TC-NER is, however, required for maintenance of genetic integrity in post-mitotic somatic cells (Lans et al., 2010). We refer the readers to excellent reviews on this pathway (Marteijn et al., 2014).

The Base Excision Repair (BER) pathway is the main pathway for repair of helix-non distorting chemically modified DNA bases. BER is less studied in *C. elegans* (Elsakrmy et al., 2020). The BER pathway is initiated by a DNA glycosylase that identify and excise certain modified bases (Figure 3). Whereas mammals have 11 DNA glycosylases there are only two DNA-glycosylases in *C. elegans*, the monofunctional uracil-DNA glycosylase (UNG-1) (Nakamura et al., 2008; Skjeldam et al., 2010) and bifunctional endonuclease III homolog NTH-1 with AP lyase activity (NTH-1) (Morinaga et al., 2009). UNG-1 repairs uracil, one of the most common types of DNA damage that arises through deamination of cytosine or misincorporation of dUMP from the nucleotide pool (Skjeldam et al., 2010). As it´s human homolog, NTH-1 repairs oxidised pyrimidines (Hazra et al., 2007). The substrate specificities of the *C. elegans* DNA glycosylases are verified only for a few characteristic substrates, and we do not know whether loss of redundancy is compensated by broader substrate specificities. UNG-1 is active on the classical UNG substrates, such as uracil and 5-hydroxymethyluracil (Papaluca et al., 2018). NTH-1 activity has been demonstrated on thymine glycol (Tg), 5-formyluracil (5-foU), and 5- hydroxymethyluracil (5-hmU) containing DNA (Morinaga et al., 2009). The lack of a DNA glycosylase dealing with oxidized purines in *C. elegans* in puzzling, but it has been suggested that RPS-3 might contribute 8-oxoG DNA glycosylase activity (Skjeldam et al., 2010). It was also reported that NTH-1 shows weak activity towards 8-oxodG paired with G, although the physiological relevance of this activity is unclear (Morinaga et al., 2009). It is also possible that oxidised pyriminines are repaired by the NER pathway in *C. elegans* (Arczewska et al., 2013). *ung-1* and *nth-1* mutant are viable, fertile with normal life span albeit with a weak mutator phenotype (Fensgård et al., 2010; Skjeldam et al., 2010; Kassahun et al., 2018). Excision of damaged bases by DNA glycosylases generates an abasic site (AP-site) that must be processed in order to generate a substrate for a DNA polymerase. The AP-site are mainly processed by AP endonucleases. EXO-3 and APN-1 are the two AP endonucleases in *C. elegans*. APN-1 harbours both 3′-diesterase and 3′-5′ exonuclease activity making it distinct from EXO-3, which lacks 3′-5′ exonuclease activity (Yang et al., 2012; Elsakrmy et al., 2020). The *exo-3* mutant animals exhibit reduced brood size and reduced lifespan (Kato et al., 2015). In contrast, the *apn-1* mutants, have normal lifespan despite having a high mutation frequency (Zakaria et al., 2010). There is no ortholog of the classical BER polymerase, DNA polymerase β. In *C. elegans* gap filling and removal of the 5’dRp end is performed by the low-fidelity DNA Polymerase θ (Asagoshi et al., 2012). Finally, BER is completed by DNA ligase I.

In *C. elegans* (Figure 3), highly deleterious double strand breaks (DSBs) are mostly repaired by error-free homologous recombination (HR) or error-prone non-homologous end joining (NHEJ) or single-strand annealing (SSA) (Lemmens and Tijsterman, 2011). The source of damage, the cell cycle phase, and the animal’s developmental stage all influence which pathway is engaged. HR is cell cycle dependent and NHEJ is cell-cycle independent (Clejan et al., 2006). The Canonical HR pathway is primarily responsible for the repair of DNA DSBs in the germline. In somatic cells, however, DSBs are mostly repaired through NHEJ. Intriguingly, loss of HR and NHEJ genes, which are lethal in mammals, are well tolerated in *C. elegans* (Lemmens and Tijsterman, 2011; Belan et al., 2021).

DNA replication errors are repaired by Mismatch Repair (MMR) (Figure 3). Orthologues of the core MMR genes; MSH-2, MSH-6, MLH-1 and PMS-2, are present in *C. elegans* (Figure 3) but *C. elegans* lacks an ortholog of MSH3 and PMS1. Thus, mismatch surveillance is mainly carried out by the MSH-2/MSH-6 heterodimer (Denver et al., 2006). RNAi-mediated depletion (Tijsterman et al., 2002) or loss of function (Meier et al., 2018) of the core MMR genes, lead to strong mutator phenotypes. As in mammals, *C. elegans msh-2* mutant showed microsatellite instability (Tijsterman et al., 2002) and reduced DNA-damage induced germline apoptosis in response to genotoxic stress (Degtyareva et al., 2002; SenGupta et al., 2013).

### *C. elegans* as a Model to Study Mechanisms of Neurodegeneration and Aging

**FIGURE 4:**
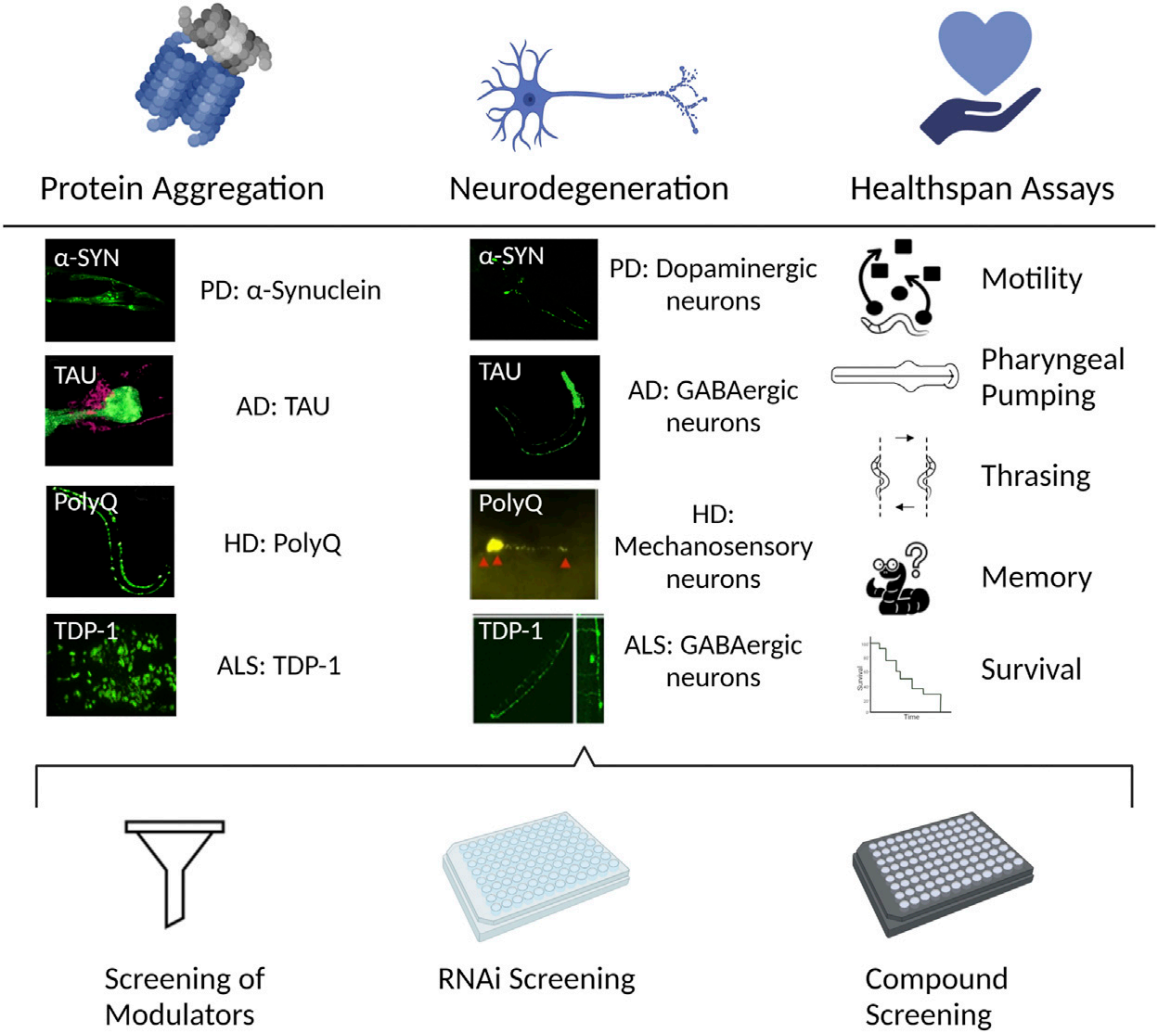
*C. elegans* to study NDs. The nematode allows for different perspectives in the study and understanding of NDs. **Left**. Protein misfolding and aggregation is one of the common features of different NDs. The transparency of *C. elegans* in combination with the use of fluorescent tags allows for the in vivo visualization of the protein aggregation process in the NDs of interest. **Center**. A consequence of the progression of NDs is the degeneration of the affected neurons. Strains can be engineered to express the aggregating proteins in the neuronal circuit of interest in the nematode, allowing to study the neurodegeneration process. **Right.** The morphological changes, featured in the different ND models like aggregation or neurodegeneration, also translate into functional challenges. The performance in the different available assays corresponds to the health status of the neuron/s of interest. **Bottom.** The above-mentioned assessments of an ND using *C. elegans* can be combined with available mutant strains for the gene/s of interest, or with screenings, either with the available existing RNAi libraries, to find genetic modulators; or compound screenings where chemical regulators of the ND progression can be discovered. The figure has been generated using Biorender.com by the author.

The advantages of *C. elegans* as a model system of aging (Figure 4) are also highly relevant for studies of NDs. In addition, the transparent body makes it possible to use fluorescent reporters (Chalfie et al., 1994) for *in vivo* visualization of the neuron(s) of interest or the whole neuronal network. The CEP neurons for example, are mechanosensory neurons responding to the neurotransmitter dopamine. *C. elegans* strains expressing Green Fluorescent Protein (GFP) in dopaminergic neurons are frequently used to study dopaminergic neuron function and integrity by live imaging. In this way, it is possible to track the integrity of neurons, but also how its state translates to a functional challenge (SenGupta et al., 2021; Palikaras et al., 2022).

The generation of RNAi libraries (Kamath et al., 2003; Rual et al., 2004) that target more than 95% of *C. elegans* genes, allows for genome-wide screens for modulators of aging and neurodegeneration (Hamilton et al., 2005; Hamamichi et al., 2008; Kuwahara et al., 2008). Although RNAi was initially found to be inefficient is neurons (Timmons et al., 2001), strains are constructed to make neurons less refractory to RNAi *e.g.* by expressing SID-1, an essential protein in the RNAi machinery, that enables intracellular transport of RNAi (Winston et al., 2002), exclusively in neurons (Calixto et al., 2010). In the same way, compound screenings can be performed in a high-throughput manner by adding the screened drugs in the media at the desired concentration/s where the worms are cultured, and analyzed. In recent years, the adaptation of gene editing methods, like the CRISPR/Cas9 system (Jinek et al., 2012), into *C. elegans* have proven again how accessible it is to adapt new techniques to the organism for easy creation and screening of tailor-made strains (Dickinson et al., 2013; Paix et al., 2015; Ward, 2015). Other available tools and methods, like the use of conditional gene expression systems or single copy transgene insertions can be found in existing reviews (Nance and Frøkjær-Jensen, 2019; Driesschaert et al., 2021).

An important strength of *C. elegans* as a model for NDs is that different subsets of neurons have been paired with assays that assess the functional status of the desired group of cells. There are methods to assess general neuronal function, like the thrashing assay, where the animals are placed in liquid media and the number of body bends while the animals “swim” are quantified (Buckingham and Sattelle, 2009). Other experiments test the locomotion or the chemotaxis of the nematode (Hosono et al., 1980; Sawin et al., 2000). On the other hand, there are assays designed to explore the health status of a specific subset of neurons. In that manner, the study of neurodegeneration is not just limited to the morphological loss of the neuron, but that process is coupled with experiments that give us an indication on the fitness status of the neurons of interest, like the basal slowing response assay, or the capacity to slow down and alter direction of movement when it reaches food, to assess dopaminergic function (SenGupta et al., 2021; Palikaras et al., 2022); or response to be touched in the head or the tail, to evaluate glutamatergic neuron status (Hart et al., 1995).

### Genomic Instability as a Driver of Aging

Many rare DNA repair diseases are modeled in *C. elegans*. *C. elegans* NER mutants phenocopy many aspects of mouse models of NER syndromes; *C. elegans* NER endonuclease ERCC-1 and XPF-1 mutants show growth arrest, developmental failure, shorter lifespan (Lans et al., 2013) and neurons are hypersensitive to UV irradiation (Sabatella et al., 2021). Loss of CSB-1 and XPA-1 in *C. elegans* result in progressive mitochondrial dysfunction (Scheibye-Knudsen et al., 2014), neurodegeneration of ALM and PLM mechanosensory neurons in response to UV irradiation (Lopes et al., 2020), and premature aging (Fang et al., 2014).

Werner syndrome (WS) is a DNA repair syndrome recognized to be a very good representation of accelerated aging. WS is caused by mutations in the *WRN* gene, encoding the WRN RecQ helicase. WS patients show early signs of aging followed by the emergence of age-related disorders such as type 2 diabetes, atherosclerosis, and osteoporosis (Lautrup et al., 2019). Malignancy and myocardial infarction are the major causes of death in these patients, as is the case in the general population, but in WS patients, the average life expectancy is only 54 years (Oshima et al., 2017). The *C. elegans* WRN-1 contains the RecQ helicase function of human WRN, but lacks the exonuclease which is encoded by a separate gene, *mut-7.* Yet, *C. elegans wrn-1* mutants domain recapitulates many phenotypes seen in the human disease; *wrn-1* mutants show shorter lifespan and impaired mitochondrial function (Fang et al., 2019b). *wrn-1* mutants also show loss of gonad cells in the distal tip of the germline but it is possible that this reflects a primary replication defect rather than a senescence - like phenotype. In an elegant recent study, plasticity of olfactory neurons was shown to depend on recruitment of epigenetic regulators through the combined activities of the MUT-7 exonuclease and the WRN-1 helicase (Hsu et al., 2021). This study is an example that shows the power of *C. elegans* to reveal novel functions and pathogenic mechanisms.

Ataxia Telangiectasia (AT) is another disease at the intersection of genetic instability, neurodegeneration, and accelerated aging. AT is caused by mutations in the *Ataxia-Telangiectasia Mutated* (*ATM*) gene, which codes for the ATM kinase. ATM was initially defined as a protein essential for DNA double-stranded break repair, but now it is recognized as a master regulator and coordinator of cellular responses to DNA damage (Shiloh and Ziv, 2013). Human AT is a pleiotropic disease with symptoms originating in a variety of physiological systems. The disease is characterized by mild immunodeficiency due to defects in cellular immunity, but also progressive cerebellar neurodegeneration (Amirifar et al., 2019). The progressive neurological manifestations are, at least in part, understood as reflecting accelerated brain aging. Other aging signs in AT children arise as they approach puberty, such as metabolic syndrome, type 2 diabetes, and osteoporosis (Shiloh and Lederman, 2017). In *C. elegans,* loss of ATM-1 is accompanied by a modest shortening of lifespan. More importantly, the *atm-1* mutants recapitulate the progressive neurodegeneration of the human disease and studies in *C. elegans* helped to pin down mitochondrial dysfunction and reduced mitophagy as a driver of this phenotype (Fang et al., 2016).

In mammals, direct induction of apoptosis signaling via the classical DDR signaling pathways appears to be a dominant mechanism driving neurodegeneration in many DNA repair diseases (Hoch et al., 2017). We recently described another mechanism driving neurodegeneration and aging using *C. elegans* as a model; We demonstrated that the loss-of-function mutation in NER (CSA-1, CSB-1 and XPA-1), ATM-1 and WRN-1 result in progressive mitochondrial dysfunction because failure to resolve constitutive DNA damage leads to depletion of the cellular NAD^+^ pool (Fang et al., 2014; Scheibye-Knudsen et al., 2014; Fang et al., 2016; Fang et al., 2019a). The activation of the DNA damage sensor poly-ADP-ribose polymerase 1 (PARP-1) is a critical event in this pathway: PARP-1 uses NAD^+^ to create poly-ADP-ribose (PAR) polymers that help to recruit the DNA damage response machinery. Constitutive activation of PARP-1 depletes the NAD^+^ pool over time. In our animal models, NAD^+^ became scarce, and enzymes that rely on NAD^+^ as a cofactor cease to function. SIR-2.1 (ortholog to mammalian SIRT1) for example, affects mitochondrial biogenesis, function, and the clearance of defective mitochondria via mitophagy. As a result, NAD^+^ deficiency causes an accumulation of damaged and malfunctioning mitochondria. Thus, in these animal models, the neurological abnormalities were indirect results of a dysmetabolic impact generated by chronic DNA damage response signaling rather than direct consequences of conventional DNA damage response signaling. Preventing NAD^+^ depletion or increasing mitochondrial biogenesis might significantly delay the development and progression of neurological symptoms using both genetic and pharmacological techniques (Fang et al., 2014; Scheibye-Knudsen et al., 2014; Fang et al., 2016; Fang et al., 2019b). To understand the effect of restoring NAD^+^ levels, multiple species (*C. elegans*, *Drosophila*, and mice) have been used to investigate the effects on the lifespan and health span of different NAD^+^ precursors (Fang et al., 2016; Schöndorf et al., 2018). In *C. elegans*, growth in the presence of 500 μM nicotinamide riboside, an NAD^+^ precursor, extended lifespan via a SIR-2.1-dependent pathway. At the same time, improvements in healthspan have also been reported including mitochondrial health, muscle strength, and motor function in different models (Belenky et al., 2007; Fang et al., 2016) (Figure 4).

### Genomic Instability as a Driver of Common Age-Related Diseases

Since *C. elegans* was introduced as a genetic model in the neurobiology field half a century ago it has provided insights into the mechanisms involving aggregation, neurodegeneration and several strains have been created to model specific human diseases (Figure 4) (Table 1).

**TABLE 1:** *C. elegans* strains used to model neurodegenerative disease.

NDD	Strain	Genotype	Tissue	Use	Reference
PD	BY273	*vtIs [Pdat-1::GFP; Pdat-1::WTα-synuclein]*	DA neurons	Expression of α-synuclein in DA neurons to assess neurodegeneration and its functional effect	Nass et al. (2002) Proc Natl Acad Sci USA 99: 3264–9
	Panneuronal-WTα-syn	*(Paex-3::α-syn; Pdat-1::GFP)*	Pan-neuronal	Panneuronal expression of WT α-syn to assess neurodegeneration and its functional effect	Lakso et al. (2003) J Neurochem 2003 July; 86 (1):165–72
	Dopaminergic-A53Tα-syn	*(Pdat-1::α-synA53T; Pdat-1::GFP)*	DA neurons	Dopaminergic expression of mutant α-syn (A53T) to assess neurodegeneration and its functional effect	Lakso et al. (2003) J Neurochem 2003 July; 86 (1):165–73
	Panneuronal-A53Tα-syn	*(Paex-3::α-synA53T; Paex-3::GFP)*	Pan-nenuronal	Panneuronal expression of α-syn (A53T) to assess neurodegeneration and its functional effect	Lakso et al. (2003) J Neurochem 2003 July; 86 (1):165–74
	UA44	*baIn11 [Pdat-1:: α-syn, Pdat-1::GFP]*	DA neurons	Expression of α-synuclein in DA neurons to assess neurodegeneration and its functional effect	Cooper et al. (2006) Science 313: 324–8
	UA49	*baIn2 [Punc-54::α-syn::GFP, rol-6 (su1006)]*	Muscle	Expression of α-synuclein in muscle to assess the aggregation process	Hamamichi et al. (2008) Proc Natl Acad Sci USA 105: 728–33
	UA50	*baInl13 [Punc-54::α-syn::GFP, Punc-54::tor-2], rol-6 (su1006)]*	Muscle	Expression of α-synuclein in muscle to assess the aggregation process but at a reduced rate due to the expresion of TOR2	Hamamichi et al. (2008) Proc Natl Acad Sci USA 105: 728–33
	NL5901	*pkIs2386 [Punc-54::alphasynuclein::YFP + unc-119 (+)]*	Muscle	Expression of α-synuclein in muscle to assess the aggregation process	van Ham et al. (2008) PLoS Genet 4: e1000027
	JVR406	*jerEx30 [ddr-2p::BiFC1 (EGFH1-LINK-SYN) + tph-1p::BIFC2 (SYN-EGFH2) + rol-6 (su1006)]*	Serotonergic neurons (tph-1p) and head, tail, ventral and dorsal nerve cords (ddr-2p)	Expression of α-synuclein in two different neural circuits expressing BiFC when there is a transfer of α-synuclein between the two neuronal populations	Tyson et al. (2017) Sci Rep. 2017 August 8; 7 (1):7506
AD	CL2355	*dvIs50 [pCL45 (snb-1::Abeta 1–42::3′ UTR (long) + mtl-2::GFP]*	Pan-neuronal	Pan-neuronal expression of hAβ3-42	Wu et al. J Neurosci. 2006 December 13; 26 (50):13102–13
	JKM2	*Is [rgef-1p::Signalpeptide-Abeta (1–42)::hsp-3(IRES)::wrmScarlet-Abeta (1–42)::unc-54 (3′UTR) +rps-0p::HygroR]*	Pan-neuronal	Pan-neuronal expression of hAβ1-42	Gallrein et al. Progress in Neurobiology 198 (2021): 101907
	UA198	*baln34 [Peat-4::Abeta42; Pmyo-2::mCherry]; adls1240 [Peat-4::GFP]*	Glutamatergic neuron	Glutamatergic neuronal expression of the hAβ1-42	Griffin et al. (2019) Dis Mod Mech 12.2 (2019): dmm037218
	BR5270	*byIs161 [rab-3p::F3 (delta)K280 + myo-2p::mCherry]*	Pan-neuronal	Pan-neuronal expression of the human tau mutant hTau [F3Δ280]	Fatuouros et al. Hum Mol Genet. 2012 August 15; 21 (16):3587–603
	CK12	*Is [aex-3::tau4R1N(P301L) + myo-2p::gfp]*	Pan-neuronal	Pan-neuronal expression of the human tau mutant hTau [P301L]	Kraemer et al. Proc Natl Acad Sci USA 100.17 (2003): 9980–9985
	FX11962	*tmIs389(Punc-119::WT hTau0N4R + Pges-1::egfp)*	Pan-neuronal	Pan-neuronal expression of the human WT tau0N4R at low level (no memory impairment)	Miyasaka et al. Front Neurosci 12 (2018): 415
	FX11974	*tmIs390(Punc-119::WT hTau0N4R + Pges-1::egfp)*	Pan-neuronal	Pan-neuronal expression of the human WT tau0N4R at high level with memory impairment	Miyasaka et al. Front Neurosci 12 (2018): 415
	UM0001	*dvIs50 [PCL45 (snb-1::Abeta 1–42::3′ UTR (long) + mtl-2::GFP]; byIs161 [Prab-3::F3 (delta)K280 + Pmyo-2::mCherry]*	Pan-neuronal	Pan-neuronal expression of hAβ1-42; hTau [F3Δ280]	Yang et al. Aging (Albany NY) 12.17 (2020): 16852
HD	ID1	*igIs1 [Pmec-3::htt57-128Q::cfp; lin-15 (+); Pmec-7::yfp]*	Mechanosensory neurons	Expression of polyQ repeats (128Q) in touch receptor neurons, resulting in extended aggregation in the mechanosensory neuronal circuit and mec phenotype	Parker et al. (2001) Proc Natl Acad Sci U S A 98: 13318–23
	ID245	*igIs245 [Pmec-3::htt57-19Q::cfp; lin-15 (+); Pmec-7::yfp]*	Mechanosensory neurons	Expression of polyQ repeats (19Q) in touch receptor neurons resulting in very low/rare aggregation in the mechanosensory neuronal circuit	Parker et al. (2001) Proc Natl Acad Sci U S A 98: 13318–23
	AM140	*rmIs132 [Punc-54::Q35::YFP]*	Muscle	Expression of polyQ repeats (35Q) in muscle. Soluble in early stages transitioning to aggregation in adult stage	Morley et al. (2002) Proc Natl Acad Sci USA 99: 10417–22
	AM141	*rmIs133 [Punc-54::Q40::YFP]*	Muscle	Expression of polyQ repeats (40Q) in muscle. Rapid aggregation since early stages showing a full aggregation phenotype in adulthood	Morley et al. (2002) Proc Natl Acad Sci USA 99: 10417–22
	AM101	*rmIs110 [Prgef-1::Q40::YFP]*	Pan-neuronal	Pan-neuronal expression of polyQ repeats (40Q) resulting in neuronal dysfunction	Gidalevitz et al. (2006), Science. March 10; 311 (5766):1471–4
	Prion-like Q25	*Ex [(myo-2p::V1Q25) + (flp-21p::Q25V2-ICR-DsRed) + pRF4 (rol-6 (su1006)]*	Pharyngeal muscle (myo-2p) and pharyngeal neurons (flp-21p)	Expression of 25Q in two different tissues expressing BiFC when there is a transfer of polyQ between the different tissues	Kim et al. (2017), Exp Neurobiol. 2017 December; 26 (6):321–328
	Prion-like Q97	*Ex [(myo-2p::V1Q97) + (flp-21p::Q97V2-ICR-DsRed) + pRF4 (rol-6 (su1006)]*	Pharyngeal muscle (myo-2p) and pharyngeal neurons (flp-21p)	Expression of 97Q in two different tissues expressing BiFC when there is a transfer of polyQ between the different tissues	Kim et al. (2017), Exp Neurobiol. 2017 December; 26 (6):321–328
ALS	Panneuronal-Human WT SOD1	*Is(Psnb-1::SOD1WT; Pmyo-2::GFP)*	Pan-neuronal	Expression of panneuronal WT human SOD1	Wang et al. (2009) PLoS Genet January; 5 (1):e1000350
	Panneuronal-Human-G85R-SOD1	*Is(Psnb-1::SOD1 (G85R); Pmyo-2::GFP)*	Pan-neuronal	Expression of panneuronal mutant G85R human SOD1 resulting in locomotor defects and aggregation	Wang et al. (2009) PLoS Genet January; 5 (1):e1000350
	Panneuronal-Human WT SOD1	*Is(Psnb-1::SOD1WT::YFP)*	Pan-neuronal	Expression of panneuronal WT human SOD1 tagged with YFP	Wang et al. (2009) PLoS Genet January; 5 (1):e1000350
	Panneuronal-Human-G85R-SOD1	*Is(Psnb-1::SOD1 (G85R)::YFP)*	Pan-neuronal	Expression of panneuronal mutant G85R human SOD1 resulting in locomotor defects and aggregation	Wang et al. (2009) PLoS Genet January; 5 (1):e1000350
	CL6049	*dvIs62 [snb-1p::hTDP-43/3′ long UTR + mtl-2p::GFP] X*	Pan-neuronal	Expression of pan-neuronal WT human TDP43	Ash et al. (2010) Hum Mol Genet. 2010 August 15; 19 (16):3206–18
	alfa-1 mutant (5x outcrossed) + GABA tag	*alfa-1 (ok3062); oxIs12 [Punc-47::GFP + lin-15 (+)]*	GABAergic neurons	Deletion mutant of alfa-1 (orthologue of human C9ORF72) with GABAergic neurons tagged. Observable degeneration of that neuronal circuit, locomotion defects and sensitive to osmotic stress	Therrien et al. (2013) PLoS One December 12; 8 (12):e83450
	GABAergic-DPR (GR)50	*Is [Punc-47::(GR)50::GFP; Pmyo-3::HIS-58::mCherry]*	GABAergic neurons	Expression of GABAergic DPR (GR)50 resulting in locomotion defects and blebbing in the GABAergic circuit	Rudich et al. (2017) Hum Mol Genet December 15; 26 (24): 4916–4928
	GABAergic-DPR (PR)50	*Is [Punc-47::(PR)50::GFP; Pmyo-3::HIS-58::mCherry]*	GABAergic neurons	Expression of GABAergic DPR (PR)50 resulting in locomotion defects and blebbing in the GABAergic circuit	Rudich et al. (2017) Hum Mol Genet December 15; 26 (24): 4916–4928
	Muscle-DPR (GR)50	*Is [Pmyo-3::(GR)50::GFP; Pmyo-3::HIS-58::mCherry]*	Muscle	Expression of muscle DPR (GR)50 resulting in locomotion defects and brood size decrease	Rudich et al. (2017) Hum Mol Genet December 15; 26 (24): 4916–4928
	Muscle-DPR (PR)50	*Is [Pmyo-3::(PR)50::GFP; Pmyo-3::HIS-58::mCherry]*	Muscle	Expression of muscle DPR (PR)50 resulting in locomotion defects and brood size decrease	Rudich et al. (2017) Hum Mol Genet December 15; 26 (24): 4916–4928

#### Parkinson’s Disease

Parkinson’s disease (PD) is the second most common ND and aging is the most important risk factor (Poewe et al., 2017; Armstrong and Okun, 2020). Loss of dopaminergic neurons in the substantia nigra region in the early stages of the disease plus formation of α-synuclein aggregates are the cardinal features of the disease (Poewe et al., 2017). Motor symptoms like trembling or coordination difficulties are characteristic but also problems in cognition, sleep or emotional stability.

In *C. elegans* dopaminergic neurons were identified already in 1975 (Sulston et al., 1975). Later, it was proposed as a PD model given its approachability, conservation of genes and pathways and available techniques and methods (Wintle and Van Tol, 2001), for example the possibility of using fluorescent proteins in the neuronal circuit of choice (Chalfie et al., 1994). The health state of dopaminergic neurons was initially addressed in neurotoxicity studies using 6-hydroxydopamine (6-OHDA) or 1-methyl-4-phenylpyridinium (MPP+) (Nass et al., 2002; Braungart et al., 2004). As worms do not have the *SNCA* gene, several groups generated strains expressing human α-synuclein in selected neuron classes (the dopaminergic circuit, in motor neurons or pan-neuronally) (Lakso et al., 2003; Cao et al., 2005; Cooper et al., 2006; Kuwahara et al., 2006) (Figure 4) (Table 1). Furthermore, variants of the *SNCA* gene that increases the misfolding tendency and assembly of α-synuclein fibrils in humans (Polymeropoulos et al., 1997; Krüger et al., 1998), like the A53T or the A30P mutant isoforms (Lakso et al., 2003; Kuwahara et al., 2006), were used to better mimic the human disease. Strains were generated expressing α-synuclein in the body wall muscles (Hamamichi et al., 2008; van Ham et al., 2008)*.* The bigger size of the cells in this tissue, made it easier to observe aggregation *in vivo*, facilitating identification of genetic modifiers of the aggregation phenotype through RNAi based screens (Hamamichi et al., 2008; van Ham et al., 2008). Newer models created to understand dynamics and specifics of PD and α-synuclein aggregation, points to misfolded α-synuclein being transferred from neuron to neuron by seeding (Desplats et al., 2009; Hansen et al., 2011). A model was created some years ago to track this α-synuclein transfer by using bimolecular fluorescence complementation (BiFC) with synaptic transmission influencing the propagation of α-synuclein (Cooper et al., 2018) (Table 1).

#### Alzheimer’s Disease

Alzheimer’s disease (AD) is the most prevalent age-related neurodegenerative disease globally, accounting for 60–70% of dementia cases. AD is marked by increasing cognitive impairment, decreased decision-making ability, behavioral abnormalities, and gradual memory loss that leads to dementia. The neuropathological features of AD are extracellular amyloid-beta (Aβ) plaques and intracellular neurofibrillary tangles (NFTs) due to hyper-phosphorylation of Tau (pTau) (Armstrong, 2006; Canter et al., 2016).

In 1998, the genome sequence of *C. elegans* revealed homologues of the AD-related proteins APP (*apl-1*) and *T*au (*ptl-1*). *C. elegans* lacks the specific features of the human APP amino acid sequence which is essential for the generation of aggregation prone Aβ peptides. *C. elegans* also lacks beta-secretase (BACE1) which generates Aβ peptides (Canter et al., 2016). To enable the use of *C. elegans* as a model for AD, Christopher Link’s group created a transgenic worm that expresses human beta-amyloid peptides, specifically Aβ3-42, in body wall muscle cells (Dosanjh et al., 2010). In this model, muscle-associated deposits accumulate. Immunostaining with the anti-Aβ antibody verified these deposits as extracellular Aβ plaques. Later a strain expressing full-length Aβ1-42 in muscle cells was made (McColl et al., 2012). In addition, a strain with pan-neuronal expression of Aβ for AD research was generated (Link, 2006). In this model, neuronal impairment was observed, such as impairments in odor-associated learning behavior, serotonin-controlled behavior, and experience-dependent learning (Dosanjh et al., 2010). NFTs formed by tau are another characteristic of AD (Strang et al., 2019) and pan-neuronal expression of Tau (normal or mutated) has been generated (Figure 4) (Table 1). Human apolipoprotein E (ApoE) is a characterized genetic predisposition marker in AD (Corder et al., 1994). Models with co-expression of distinct human ApoE alleles and an Aβ peptide was created to provide an *in vivo* platform to explore Aβ-associated neurotoxic effects in distinct glutamatergic neurons (Griffin et al., 2019) and serotonergic hermaphrodite specific neurons (HSNs) (Sae-Lee et al., 2020). Combined with machine learning and other laboratory techniques, the *C. elegans* AD model has been used as a model system in drug development (Xie et al., 2022).

### Huntington’s Disease

Huntington’s disease (HD) is a neurodegenerative disorder caused by dominantly inherited glutamine repeats (polyQ) in the huntingtin gene (*HTT*). The product of *HTT* is the protein huntingtin (HTT). These glutamine repeats, become pathogenic when the number of repeats reach 36 (Tabrizi et al., 2020). Then, HTT folds abnormally, forming aggregates that disrupt cellular functions and result in cell death. The progression of the disease causes loss of control of voluntary and involuntary movements, and dysfunction in cognition and behavior.

Early efforts to use *C. elegans* as a model for HD included the assessment of sensory neurons by dye filling assays and the impact of endogenous expression of diverse lengths of polyQ repeats in aggregation, neurodegeneration and response to stimulus (Faber et al., 1999). Several models were established expressing different lengths of polyQ repeats in different tissues, from body wall muscle (Satyal et al., 2000; Morley et al., 2002) to mechanosensory neurons (Parker et al., 2001). When expressed in body wall muscle, tracking the process of polyQ aggregation was easy. Thus, it was possible to study how the different tracts of polyQ assembled at different rates, and it became evident that the motility of the nematode was affected in a directly proportional way as the organism aged. When expressed in touch receptor neurons, the observable aggregation was accompanied with defects in the response to touch, especially in the tail, accompanied by abnormalities in the neuronal processes. It is also important to remark that the effect of polyQ aggregation did not impact only the expressed tissue of its function, but altered and deregulated other processes, like the heat shock response (Satyal et al., 2000). The latter illustrates how the small animal system can reveal layers of insight in a way that cellular model system cannot (Figure 4) (Table 1).

These studies provided the tools necessary for carrying out screenings for modifiers of polyQ aggregation (Nollen et al., 2004; Voisine et al., 2007) and show how the presence of misfolded proteins affect the cellular environment (Holmberg et al., 2004; Gidalevitz et al., 2006). An advantage of this system is the difference in rate of aggregation between the strains with different number of repeats, which bypasses the problem of the lack of expansion of the polyQ tracts in *C. elegans*. In that way, one could use a strain with a low rate of aggregation and try to find a modifier, genetic or drug that increases aggregation. On the other hand, it is possible to use strains that have a higher level of aggregation in order to find modifiers that would decrease it. The possibility of using strains that produce misfolded proteins also allows to study regulation and processes like the unfolded protein response (UPR) or ER-associated degradation (ERAD), not just being useful for HD research but also to the understanding of proteostasis in general (Silva et al., 2011; Muñoz-Lobato et al., 2014). After studies suggesting the transmission of polyglutamine aggregates between cells as the mechanism for the progress of the disease (Ren et al., 2009; Pecho-Vrieseling et al., 2014; Pearce et al., 2015), a strain was engineered to assess the transmission of polyQ between different group of cells by BiFC, with neurodegenerative phenotypes increasing with the length of the polyQ tract (Kim et al., 2017).

### Amyotrophic Lateral Sclerosis

Amyotrophic lateral sclerosis (ALS) is a common degenerative motor neuron disease, impacting the motor cortex, brain stem, and spinal cord (Ragagnin et al., 2019). Mutations in four genes, *SOD1, C9ORF72, TARDBP*, and *FUS/TLS* cause the majority of cases. The disease is manifested by progressive locomotor impairment and weakness, speech disturbance and pulmonary complications that lead to respiratory failure and death (Zarei et al., 2015).

*C. elegans* has been used to model pathology emanating from the specific mutations; for example, expression of mutant isoforms of human SOD1 systemically induced by heat shock or in different tissues muscle (Oeda et al., 2001), motor neurons (Witan et al., 2008) or pan-neuronally (Wang et al., 2009), result in proteotoxicity and/or locomotion defects. *C. elegans* has an ortholog of *C9ORF72*, *alfa-1,* and the null mutant evidenced locomotion problems, suffered neurodegeneration in GABAergic neurons and was sensitive to osmotic stress (Therrien et al., 2013)*.* An intronic hexanucleotide repeat (GGGGCC) in *C9ORF72* was identified as the most common genetic variant in familial ALS (DeJesus-Hernandez et al., 2011; Renton et al., 2011). Translation of this transgene resulted in the production of toxic dipeptide repeats proteins (DPRs) (Rudich et al., 2017), as seen in the human disease. When expressed in motor neurons, this resulted in neurodegeneration and motility defects. When expressed in muscle, the motility defects were accompanied by reduction in brood size and developmental defects.

Other efforts have focused on *TARDBP* and *FUS*. The products of both genes, TDP-43 and FUS, respectively, are DNA/RNA binding proteins involved in transcription and splicing regulation (Mackenzie et al., 2010). Mutants of the *C. elegans* homologues of these genes*, tdp-1* and *fust-1*, respectively, suggest functional conservation. Lack of TDP-1 induces accumulation of double stranded RNA (dsRNA), genomic instability and changes in the chromatin organization (Saldi et al., 2014; Saldi et al., 2018; Mitra et al., 2019), accompanied by locomotion defects (Zhang et al., 2012) and sensitization to osmotic and oxidative stress (Vaccaro et al., 2012b). Loss of FUST-1 suppressed miRNA-mediated gene silencing (Zhang et al., 2018) and dysregulated circRNA formation (Cao, 2021). When expressing human TDP-43 in a pan-neuronal manner, motility defects were observed (Ash et al., 2010). Interestingly, pan-neuronal expression of human FUS did not cause any observable defect (Murakami et al., 2012). However, expression of disease-causing mutations of TDP-43 and FUS both caused neurodegeneration and locomotion defects when expressed pan-neuronally (Murakami et al., 2012; Liachko et al., 2013) or in the GABAergic circuit (Vaccaro et al., 2012a) (Figure 4) (Table 1). Defective DNA damage response has been linked to many subtypes of ALS (Guerrero et al., 2016). Recently, in an *C. elegans* ALS model, it was shown that TDP-43 is recruited in DSB site and plays a vital role in TDP-43 driven pathology (Mitra et al., 2019). Overexpression of another DNA damage response modulator, FUS, in *C. elegans* leads to defective neuro muscular signalling and motor defects (Markert et al., 2020). Loss of RAD-23, a homolog of the human HRAD23A and HR23B proteins that serve as accessory proteins in the damage recognition step of NER and in coupling of NER-mediated DNA repair to the ubiquitin/proteasomal system (UPS), protects motor neurons and improves movement disorders by enhancing the clearance of TDP-43 and SOD-1 aggregation in a *C. elegans* ALS model (Jablonski et al., 2015).

### Perspectives

Evolutionarily *C. elegans* is distant from humans. It lacks organ systems, like the brain, liver, kidney, blood, and a blood-brain barrier. Hence, there are some relevant constraints for the use of this model research into the intersection of genome maintenance, aging and neurodegeneration. To begin with, it feeds on bacteria, making biochemistry inherently difficult. Secondly, despite possessing 959 somatic cells, investigating somatic stem cell biology is difficult (Sulston and Horvitz, 1977). Thirdly, *C. elegans* lacks adaptive immunity. Fourthly, *C. elegans* lacks DNA methylation, which is critical in aging and neurodegeneration (Corsi et al., 2015). Finally, achieving proper sample sizes for biochemical assays such as immunohistochemistry, immunoprecipitation, chromatin immunoprecipitation, microarray, RNA and DNA sequencing requires whole animal extract, which may mask effects in certain cell types.

**FIGURE 5:**
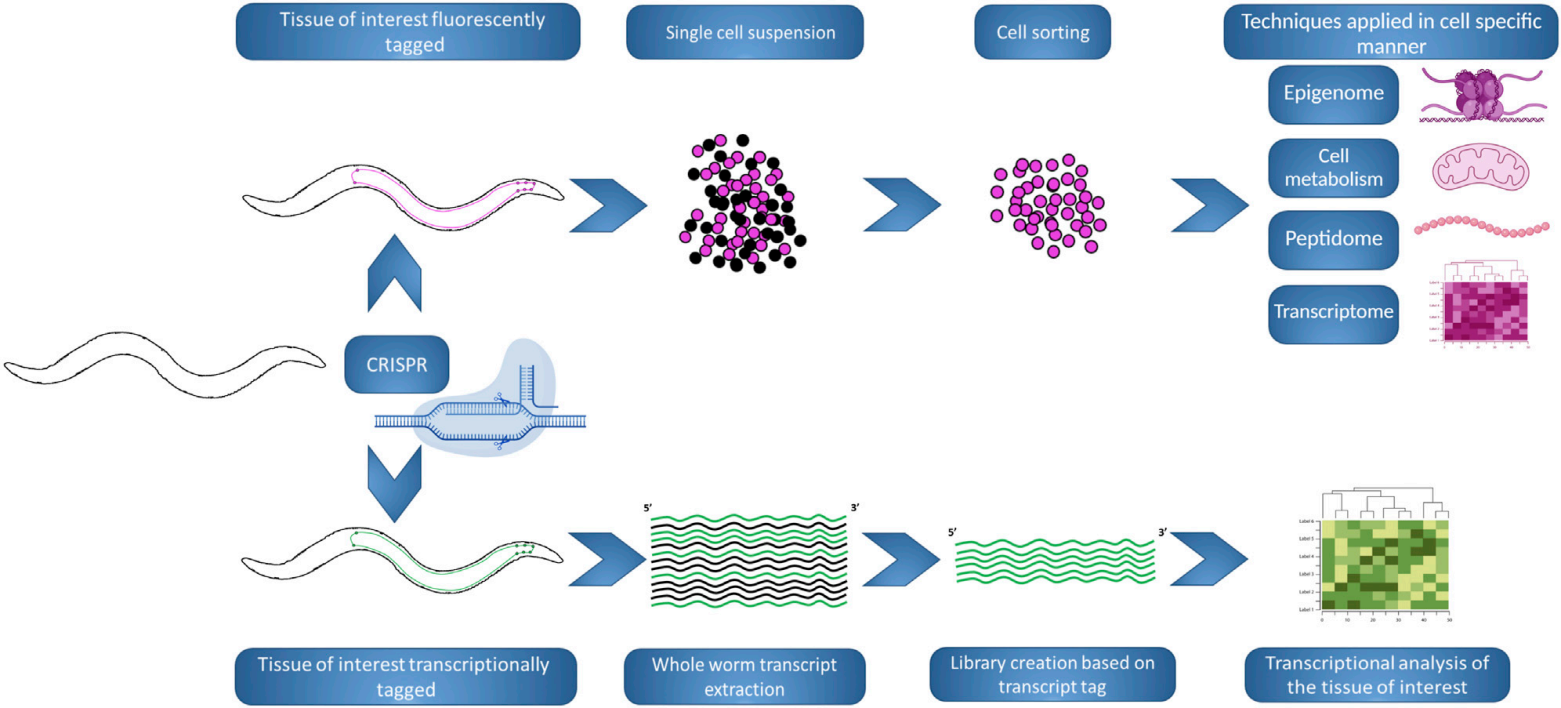
Innovative workflows using *C. elegans* to study NDs. The development of new workflows and or techniques in the nematode allows for news scopes in the study of NDs, allowing to switch between whole-organism to single cell perspectives, at one’s convenience. **(Top).** A strain can be generated by CRISPR in order to tag with a fluorescence marker (pink) the tissue of interest. Posteriorly, large synchronized populations of the strain can be raised and chemically digested, in order to obtain a cell suspension that be FACS sorted based on the fluorescence tag. The resulting sorted population can be used in different analyses and techniques, where small variations could be masked if analysing whole worms. **(Bottom).** A strain can be generated by CRISPR in order to tag biochemically (green) the transcripts produced in the tissue of interest. Posteriorly, large synchronized populations of the strain can be raised and whole organism extraction is performed, but the creation of the transcript library will be biased by the presence of the biochemical tag. Following transcriptional analysis will inform specifically about changes in the tissue of interest. The figure has been generated using Biorender.com by the author.

Despite these obvious limitations, *C. elegans* has been used as a model to study NDs for decades (Figure 5). But what at a glance could be taken as a disadvantage, is actually one of its main strengths, there are numerous possibilities for adaptation to different studies, techniques and scopes. This potential remains to this day, with many new methods that allow for more specific studies of the neuronal milieu of the nematode. Most remarkably, in the past few years, the common effort of different *C. elegans* labs led to the creation of the CeNGEN project (Hammarlund et al., 2018), a consortium formed with the goal of identifying the neuronal gene expression of the nematode with single cell resolution (Taylor et al., 2021) and that was made available online (https://cengen.shinyapps.io/CengenApp/). This work built upon previous work consisting of the creation of strains with the neuron/s of interest tagged with a fluorescent reporter, culturing big quantities of the desired strain and the posterior digestion and processing of the animals in order to obtain a suspension of cells. Lastly, the fluorescent cells would be sorted using FACS and, in this case, the pipeline continued with RNA-seq in order to analyze their gene expression (Spencer et al., 2014). These methods permit to limit a study to the desired neuronal group. Nonetheless, an adult *C. elegans* hermaphrodite has 959 somatic cells of which 302 are neurons; which means that subtle changes in neuronal gene expression could be masked, plus it enables to spot differences between the different groups of neurons. Furthermore, although the downstream step in the mentioned publications was gene expression studies, when the desired cell population is sorted, the possibilities for techniques and methods are almost infinite. The study and identification of neuropeptides (Van Bael et al., 2018) or the measurement of oxygen consumption rate to assess mitochondrial function (Koopman et al., 2016), would become specific to the population of neurons of interest; and it is obvious how this can impact and be revolutionary in the study of NDs on *C. elegans.*

Different approaches have been used for bypassing the possible masking of changes in the neuronal expression by analyzing whole animals. Other labs generated methods to tag the transcripts of the desired tissue. In (Ma et al., 2016), the muscle transcriptome was analyzed by tagging the spliced leader (SL) RNA gene using a muscle specific promoter, allowing to process whole animals collecting total RNA, but then focusing their study in the transcripts carrying the SL tag. Of course, this method could be adapted to other tissues, being possible to exploit in neuronal subsets. In (Alberti et al., 2018), cell type specific microRNAs are methylated by expressing a plant specific methyltransferase in the desired subset of neurons, and biasing the cloning and creation of the library for RNA-seq in the existence of methylation, permitting cell specific resolution. This work does not highlight just the importance of neuron specific expression, but also the study of other RNA families, that although not generating protein products, are very important in the regulation and therefore the metabolism and functionality of a cell. With that in mind, the study of circular RNAs, a fraction of RNAs that lack 5′ and 3′ ends, may become of importance in the NDs research on *C. elegans*. Circular RNAs are the products of pre-mRNA transcripts that undergo backsplicing events. Although their functions are not fully understood, they are involved in regulation of gene expression, therefore, being potential modulators of numerous processes in the cell. They are exceptionally stable and thought to accumulate during the aging process (Cortés-López et al., 2018). They could become a major factor to understand and assess the fitness of a neuron. Additionally, small RNAs resulting from transfer RNAs (tRNA), tRNA-derived fragments (tRFs), are also accumulated as the organism ages, being potential candidates as aging biomarkers (Shin et al., 2021).

These approaches, which provide a more subtle understanding of the transcriptional context of the neurons, can be complemented with a series of new fluorophores with enhanced brightness. This increase in brightness makes it possible that lowly expressed genes can still be visualized *in vivo* in the worm. Examples like mNeonGreen or mScarlet (Shaner et al., 2013; Bindels et al., 2017) have appeared as alternatives to GFP or mCherry to label proteins that are not highly expressed, as in (Hostettler et al., 2017), where mNeonGreen consistently outperforms the levels of brightness of GFP.

*C. elegans* became the first organism with a described global description of its neural circuit or connectome (Cook et al., 2019; Alicea, 2020). Understanding the neuronal circuit as a dynamic network, that adapts to challenges as the case of a NDs model, may help us understand the adaptations the neuronal circuit goes through as a whole when subsets of neurons start the process of neurodegeneration. This could be combined with approaches like NeuroPAL, a *C. elegans* strain created where every single neuron can be identified based on its fluorophore colour and position in the neuronal circuit (Yemini et al., 2021), allowing to track whole-brain neuronal dynamics and activity. Furthermore, the NeuroPAL strain can be used for identifying neuronal gene expression or the effect of mutations in neuronal fate and development.

Other efforts focus on facilitating the way of carrying out assays in the field. The previously mentioned assays, although accessible and affordable, may require time consuming tedious tasks such as counting aggregates, body bends or paralyzed animals. Trying to ease these procedures by automating them, would make the assessment of NDs models on *C. elegans* an easier task and would permit to assess more strains or conditions in the same assay, potentiating one of the advantages of the organism, the possibilities for high-throughput applications. The creation of systems like the WorMotel (Churgin and Fang-Yen, 2015), where assayed worms are cultured in multiwell plates and images of them are taken in the desired interval of times, allowing to discern their movement. Systems like this allow us to determine parameters like locomotion, lifespan and behavior (SenGupta et al., 2021). Other groups have developed methods to automatize the quantification of aggregate formation (Molenkamp et al., 2021; Vaziriyan-Sani et al., 2021) or the number of head thrashes (Zhang et al., 2022); both methods that have been described previously as traditional ways to analyze ND models (Figure 5). The continuous development and improvement of the existing methods and the automatization of traditionally laborious assays will allow for more precise and wide analysis; doing possible the inclusion of new variables or scopes in this kind of assays.

## Conclusion

DNA damage and its repair are generally linked to the aging process. Effective studies of the interactions between these processes depends on a model system that allows physiological aging to be taken into account. Although some studies have emerged where *C. elegans*, with its simplified DNA repair mechanisms have been studied in light of the described aging pathways, there is still much that remains to be explored and we are probably only scratching the surface on the information that can be gained. Genetic interactions of different DNA repair pathways have been demonstrated*, e.g.* between the BER and NER pathway (Arczewska et al., 2013) but systematic studies to elucidate the extent of the collaboration between different DNA repair pathways are warranted. The segmental progeroid and other DNA repair deficiency syndromes have a plethora of phenotypes from different organs and organ systems and *C. elegans* serves as powerful model to understand the response and integration to different kinds of DNA repair pathways in different organ systems. Moreover, *C. elegans* has the potential to reveal how DNA repair impact on the induction of senescence, telomere attritions and possibly other, yet to be defined, mechanisms of aging and how different processes might operate simultaneously and are integrated. The recent identification of involvement of MUT-7/WRN-1 regulating neuronal plasticity (Hsu et al., 2021) is one example where *C. elegans* may have revealed a new mechanism. At present is not known, for example, whether non-canonical MMR, where MMR is uncoupled from DNA replication, may play a role in *C. elegans* neurons.

Another observation that has been made in *C. elegans* is that although DNA repair in general is protective, loss of function in individual DNA repair enzymes may be associated with beneficial phenotypes: A hypomorphic mutation of ATR in humans causes accelerated aging disease known as Seckel syndrome. The *C. elegans* ATL-1 is orthologous to human ATR. Interestingly, *atl-1* mutants exhibit extended life span, perhaps as a consequence of mild oxidative stress and mitochondrial dysfunction (Suetomi et al., 2013). We recently showed that removing the BER DNA glycosylase NTH-1 protected against age dependent loss of dopaminergic neurons in a *C. elegans* PD model. These animals showed significantly less accumulation of oxidative lesions and lethal SSBs in nuclear and mitochondrial DNA. The lack of NTH-1 prevented the accumulation of BER intermediates, which activated mitochondrial genotoxic stress and mitohormesis, altogether orchestrating a response that protects DA neurons from α-synuclein-induced neurotoxicity (SenGupta et al., 2021). Future work will show whether similar mechanisms compensating for loss of DNA repair activity are operating also in mammals.

Thus, the combination of approachability, versatility and decades of accumulated knowledge, from the very early days of genetic studies screening for phenotypes and measuring maximum lifespan; have led us to an exciting moment in the field. The new genetic tools and novel technologies, like single neuron resolution transcriptional studies or assay automatization, expand the existing traditional methods and allow for wider and newer scopes where we will be able to gain a deeper understanding of the role of DNA repair in NDs, maybe permitting us 1 day to expand our healthspan much closer to our lifespan.
